# The Octopus and the Independent Press

**Published:** 1908-05

**Authors:** 


### The Octopus and the Independent Press.

In an editorial on "Another Victim of the Octopus" Dr. Register, of the *Charlotte Medical Journal,* after describing the killing off of a certain journal, says:

"Succinctly and briefly that is the whole situation in a nutshell: the result of the acknowledged policy of the *Octopus,* as Brother Daniel of the Texas "Red Back" has been pleased to term the national organization, to squeeze out the independent journals and to force upon the profession a medical journalism whose very life blood is crushed out, a press which possesses no independence of thought and which in no way represents the great mass of the medical fraternity. Years ago, when the American Medical Association, during the period of its reorganization, was a toddling infant, unable to stand alone, voiceless and helpless, it was largely with the help of the independent medical press of the country that this association took its first uncertain tottering steps. Independent journals lent their aid and by their widespread advocacy of medical organization nursed the foster-child until it became a healthy, growing youth. By and by we see the American Medical Association grown to proportions embracing the whole of the United States with a weekly medical journal which is sent to every member of the Association upon the payment of his annual society dues. No longer does this ungrateful child require the kindly care of its foster-mother; having no further use for independent medical journals it turns upon and seeks to devour them, destroy them, force them out of existence.

"No society journal can be untrammelled and in the highest degree helpful in the same sense that independent journals are, for the reason that editors of the former hold their office by virtue of their ability to conciliate, to please the faction which may happen to be in power. Censorship of the press permits the publication of matter—good, bad, or indifferent—which happens not to conflict with the views of the 'powers that be.' Society journals do not correctly represent the whole medical profession, but rather a faction or clique which holds the reins.

"However, while we believe that we have enumerated some of the grave faults of organization journals, what we wished to do was to point out some of the reasons why independent journals find it increasingly difficult to exist. When a physician becomes a member of the American Medical Association he pays his annual dues, amounting to $5. He then becomes a subscriber, without additional expense to the *Journal of the Americal Medical Association*, which comes to him every week. He may also be a member of a State society, which likewise has its own journal. As a member, therefore, of his State society, he is also a subscriber to a second journal, which may come to him weekly, or, it may be, monthly. Again, in a few instances, county societies possess official organs to which members become subscribers by virtue of having joined the society and paid up their dues. Thus the physician gets regularly several journals and the vast majority of doctors could not find time to read more. He finds the society journals are quite as much as he can find time to 'look over.'

"The avowed purpose of the American Medical Association's policy is to so perfect the organization of the whole country that upon joining the county society the initiate at the same time automatically becomes a member both of his State and of the National Associations. Payment of the fees of each of these organizations also puts him on the list of subscribers to the several journals of the respective societies. It is also the purpose of the *Octopus,* if this plan of organization succeeds, to have at least every State society establish its official periodical. As this plan is successfully carried out the independent journals find their subscribers falling off, not because they are finding their organization journals better, but because they can not afford and can not find time to read so many. He simply has no choice; he *must* become a subscriber when he becomes a member of the organization [whether he wishes to or not].

"Now, we have no quarrel with organization; it has done more to uplift the medical profession than has any other thing in all time. But it is this system that we condemn. If the so-called society journals were placed on the same footing with the so-called independent journals—allowing a member of the associations to subscribe or not as he might choose—there would exist some element of fairness in the competition.

"Make the society dues and the subscription to the official journal separate and distinct, allow the doctor the privilege of choosing his medical journal, and both can live. Merit should be the test.

The existence of an independent medical press is the only means by which a healthy balance can be preserved against the autocratic and bureaucratic tendencies of rampant organization, and whether this check is to be thrown off or preserved only the future can decide."

Dr. Register then quotes another writer as follows:

"And do you know that, if independent journalism were nonexistent, any physician who dared to criticize or make himself obnoxious to the powers that be, would have a very hard and miserable life of it. He would be unable to have any of his articles, even of a purely scientific character, accepted by any journal; his books and other writings would be unfavorably commented upon or he would be killed by silence. This is not a mere phantasmagoria; I have simply filled in and completed the picture, an outline of which can be seen even now by those of strong discernment. That not a word of criticism of the management, that no expose of machine politics, etc., could ever be published, goes without saying. In short, we would have a medical bureaucracy, strong, irresponsible, unscrupulous and comparable only to the Russian bureaucracy before the advent of the Duma. Wouldn't that be the greatest calamity that could befall the medical profession?"

